# Rivaroxaban With or Without Aspirin in Patients With Heart Failure and Chronic Coronary or Peripheral Artery Disease: The COMPASS Trial

**DOI:** 10.1161/CIRCULATIONAHA.119.039609

**Published:** 2019-06-05

**Authors:** Kelley R. Branch, Jeffrey L. Probstfield, John W. Eikelboom, Jackie Bosch, Aldo P. Maggioni, Richard K. Cheng, Deepak L. Bhatt, Alvaro Avezum, Keith A.A. Fox, Stuart J. Connolly, Olga Shestakovska, Salim Yusuf

**Affiliations:** 1 Cardiology Division, University of Washington, Seattle (K.R.B., J.L.P., R.K.C.).; 2 Population Health Research Institute, McMaster University and Hamilton Health Sciences, ON, Canada (J.W.E., J.B., S.J.C., O.S., S.Y.).; 3 National Association of Hospital Cardiologists Research Center (ANMCO), Firenze, Italy (A.P.M.).; 4 Brigham and Women’s Hospital Heart and Vascular Center, Harvard Medical School, Boston, MA (D.L.B.).; 5 Dante Pazzanese Institute of Cardiology and Hospital Alemão Oswaldo Cruz, São Paulo, Brazil (A.A.).; 6 Centre for Cardiovascular Science, University of Edinburgh, Scotland (K.A.A.F.).

**Keywords:** aspirin, cardiovascular diseases, coronary artery disease, heart failure, peripheral artery disease, randomized controlled trial, rivaroxaban

## Abstract

**Background:**

Patients with chronic coronary artery disease or peripheral artery disease and history of heart failure (HF) are at high risk for major adverse cardiovascular events. We explored the effects of rivaroxaban with or without aspirin in these patients.

**Methods:**

The COMPASS trial (Cardiovascular Outcomes for People Using Anticoagulation Strategies) randomized 27 395 participants with chronic coronary artery disease or peripheral artery disease to rivaroxaban 2.5 mg twice daily plus aspirin 100 mg daily, rivaroxaban 5 mg twice daily alone, or aspirin 100 mg alone. Patients with New York Heart Association functional class III or IV HF or left ventricular ejection fraction (EF) <30% were excluded. The primary major adverse cardiovascular events outcome comprised cardiovascular death, stroke, or myocardial infarction, and the primary safety outcome was major bleeding using modified International Society of Thrombosis and Haemostasis criteria. Investigators recorded a history of HF and EF at baseline, if available. We examined the effects of rivaroxaban on major adverse cardiovascular events and major bleeding in patients with or without a history of HF and an EF <40% or ≥40% at baseline.

**Results:**

Of the 5902 participants (22%) with a history of HF, 4971 (84%) had EF recorded at baseline, and 12% had EF <40%. Rivaroxaban and aspirin had similar relative reduction in major adverse cardiovascular events compared with aspirin in participants with HF (5.5% versus 7.9%; hazard ratio [HR], 0.68; 95% CI, 0.53–0.86) and those without HF (3.8% versus 4.7%; HR, 0.79; 95% CI, 0.68–0.93; *P* for interaction 0.28) but larger absolute risk reduction in those with HF (HF absolute risk reduction 2.4%, number needed to treat=42; no HF absolute risk reduction 1.0%, number needed to treat=103). The primary major adverse cardiovascular events outcome was not statistically different between those with EF <40% (HR, 0.88; 95% CI, 0.55–1.42) and ≥40% (HR, 0.81; 95% CI, 0.67–0.98; *P* for interaction 0.36). The excess hazard for major bleeding was not different in participants with HF (2.5% versus 1.8%; HR, 1.36; 95% CI, 0.88–2.09) than in those without HF (3.3% versus 1.9%; HR, 1.79; 95% CI, 1.45–2.21; *P* for interaction 0.26). There were no significant differences in the primary outcomes with rivaroxaban alone.

**Conclusions:**

In patients with chronic coronary artery disease or peripheral artery disease and a history of mild or moderate HF, combination rivaroxaban and aspirin compared with aspirin alone produces similar relative but larger absolute benefits than in those without HF.

**Clinical Trial Registration:**

URL: https://www.clinicaltrials.gov. Unique identifier: NCT01776424.

## Abstract 2

Supplemental Digital Content is available in the text.

**Clinical Perspective**

**What Is New?**

- The COMMANDER HF trial (A Study to Assess the Effectiveness and Safety of Rivaroxaban in Reducing the Risk of Death, Myocardial Infarction, or Stroke in Participants with Heart Failure and Coronary Artery Disease Following an Episode of Decompensated Heart Failure) demonstrated that in patients with coronary artery disease, low ejection fraction (≤40%), and recent heart failure exacerbation, low-dose rivaroxaban treatment did not improve major adverse cardiovascular events, although thrombotic outcomes were reduced.
- In participants in the COMPASS trial (Cardiovascular Outcomes for People Using Anticoagulation Strategies) with a history of mild to moderate heart failure (exclusion criteria included left ventricular ejection fraction <30% and New York Heart Association functional class III and IV heart failure), combination rivaroxaban 2.5 mg BID and aspirin compared with aspirin alone demonstrated consistent relative risk reduction but higher absolute risk reduction for major adverse cardiovascular events and mortality compared with those without heart failure.

**What Are the Clinical Implications?**

- Patients with a history of mild to moderate heart failure and chronic atherosclerotic disease are a high-risk population, and the addition of low-dose rivaroxaban 2.5 mg BID to aspirin results in a similar relative but higher absolute risk reduction in major adverse cardiovascular events and mortality compared with those without heart failure.
- In COMPASS trial patients with a decreased ejection fraction (≤40%), the higher cardiovascular mortality outnumbers the antithrombotic benefits of low-dose rivaroxaban and is directionally consistent with the neutral findings in the COMMANDER HF trial.

**Editorial, see p 538**

Most patients with heart failure (HF) have concomitant coronary artery disease (CAD),^1^ which can lead to worsening HF through myocardial ischemia or infarction and can also predispose to adverse cardiovascular events. Patients with CAD or peripheral artery disease (PAD) who also have HF have nearly a 2-fold higher risk of subsequent cardiovascular events than those without HF despite contemporary medical therapy that typically includes aspirin.

The COMMANDER HF trial (A Study to Assess the Effectiveness and Safety of Rivaroxaban in Reducing the Risk of Death, Myocardial Infarction, or Stroke in Participants with Heart Failure and Coronary Artery Disease Following an Episode of Decompensated Heart Failure) tested whether patients with chronic CAD, a reduced ejection fraction (EF; <40%), and a recent (<1 month) acute hospitalization for HF would benefit from the addition of rivaroxaban 2.5 mg BID to contemporary medical therapy.^2^ Rivaroxaban did not reduce the primary outcome, a composite of stroke, myocardial infarction (MI), or all-cause mortality. In contrast, the COMPASS trial (Cardiovascular Outcomes for People Using Anticoagulation Strategies) demonstrated that in patients with chronic CAD and PAD, the combination of rivaroxaban 2.5 mg twice daily and aspirin 100 mg once daily reduced the relative risk of stroke, MI, or cardiovascular death (major adverse cardiovascular events [MACE]) by 24% compared with aspirin.^3^ Unlike COMMANDER, COMPASS excluded patients with recently decompensated HF or severe HF as defined by baseline EF of ≤30% or New York Heart Association functional class III or IV HF. In the present report, we explore the effects of rivaroxaban with or without aspirin on MACE and bleeding in COMPASS patients with or without a history of HF and according to left ventricular EF recorded at baseline.

## Methods

COMPASS (ClinicalTrials.gov NCT01776424) is a multicenter, double-blind, randomized, placebo-controlled trial of 27 395 stable patients with chronic CAD and PAD comparing rivaroxaban 2.5 mg twice daily plus aspirin 100 mg once daily or rivaroxaban 5 mg twice daily to aspirin 100 mg once daily (rivaroxaban arm).^4^ The primary outcome was a composite of cardiovascular death, stroke, or MI, and the main safety outcome was a modification of the International Society of Thrombosis and Haemostasis (ISTH) major bleeding criteria. The trial design and inclusion and exclusion criteria have been reported previously. This included methods for randomization to pantoprazole or placebo for patients who were not taking a proton pump inhibitor at baseline.^4^ Human subjects approval was obtained for each center, and written informed consent was obtained from all participants. Patients with severe HF with known left ventricular EF <30% or New York Heart Association functional class III or IV symptoms and those requiring oral anticoagulation or dual-antiplatelet therapy were excluded. A history of atrial fibrillation was not recorded at the time of randomization. A history of HF at randomization was determined by the clinical site and included both preserved and reduced EF. No further criteria or documentation were required. Baseline left ventricular EF was recorded when available but was not an inclusion requirement for COMPASS.

The data that support the findings of this study are available from the corresponding author on reasonable request, although anonymized data and materials will be made publicly available in the near future. As part of a preplanned subanalysis, we report the effects of randomized treatments in patients with or without a history of or current HF at baseline and according to EF at baseline (<40%, ≥40%, or no EF data available) on the primary outcome of MACE, MACE plus HF hospitalization during the trial, mortality, and major bleeding.^4^ We defined HF with reduced EF (HFrEF) as EF <40% and HF with preserved EF as EF ≥40%. Net clinical benefit was defined as the primary efficacy outcome plus severe bleeding (fatal bleeding or bleeding into a critical organ), as reported previously.^3^

### Statistical Analysis

Analyses were conducted according to the intention-to-treat principle. We compared baseline characteristics of patients with and without HF at baseline using Wilcoxon 2-sample tests for continuous variables and Pearson χ^2^ tests for categorical variables. Survival analyses were based on the time to a first event. Patients could have >1 event, but we counted only the first event. We separately compared each of 2 rivaroxaban-based regimens with the aspirin-only control group using stratified log-rank tests. The stratum variable was treatment with proton pump inhibitor at baseline: not randomized to proton pump inhibitor, randomized to active pantoprazole, or randomized to pantoprazole placebo. We estimated hazard ratios (HR) and corresponding 95% CIs using Cox proportional hazards models stratified by treatment with proton pump inhibitor at baseline. The assumption of the proportional hazards was verified using the plots of log of the negative log of survival function against the log of time. A 2-sided *P* value <0.05 was considered significant. There was no correction for multiple comparisons. All data were housed and analyzed at the Population Health Research Institute in Hamilton, Ontario, Canada, independently from the sponsor. Analyses were performed with SAS software for Linux, version 9.4 (SAS Institute Inc, Cary, NC).

## Results

Baseline characteristics of the trial population are shown in Table 1. Of the 27 395 patients enrolled in COMPASS, 5902 (22%) had a history of HF at baseline. Left ventricular EF was available in 16 792 patients (61.3%), including 4971 of 5902 (84.2%) of those with HF. Patients with HF were younger, were more likely to be Eastern European, had a higher rate of current smoking, and were more likely to have a history of MI (Table 1). Patients with HF were also more often treated with an angiotensin-converting enzyme inhibitor or angiotensin receptor blocker, diuretic, β-blocker, and lipid-lowering agent than patients without HF (Table 1).

**Table 1.**
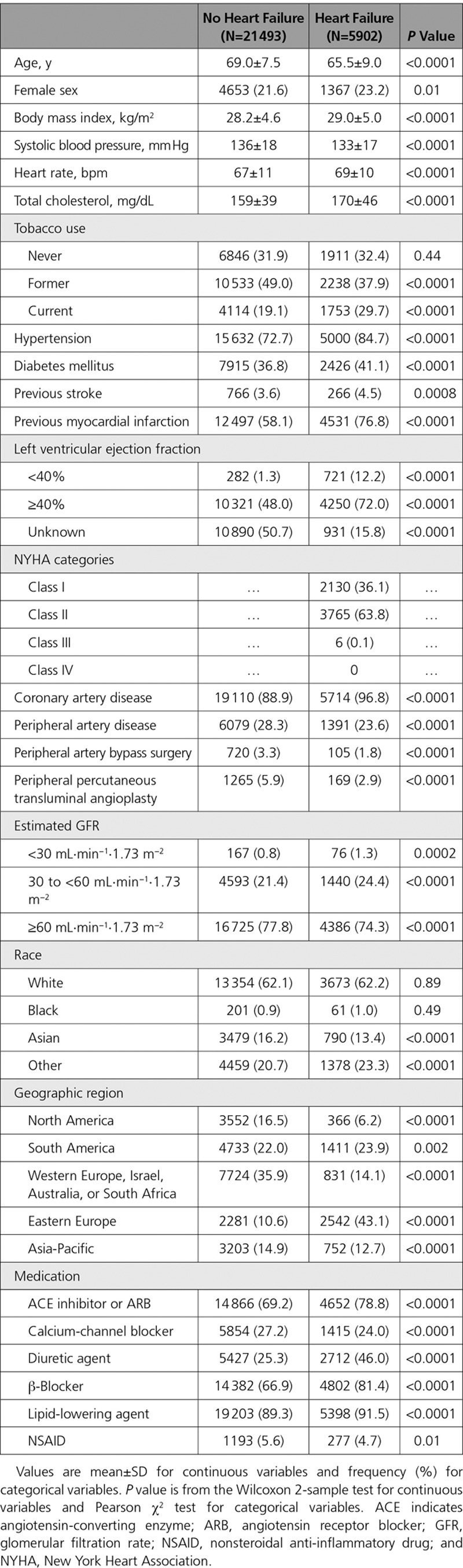
Baseline Characteristics of Patients With or Without a History of Heart Failure at Baseline

### HF and Outcomes

**Figure 1.**
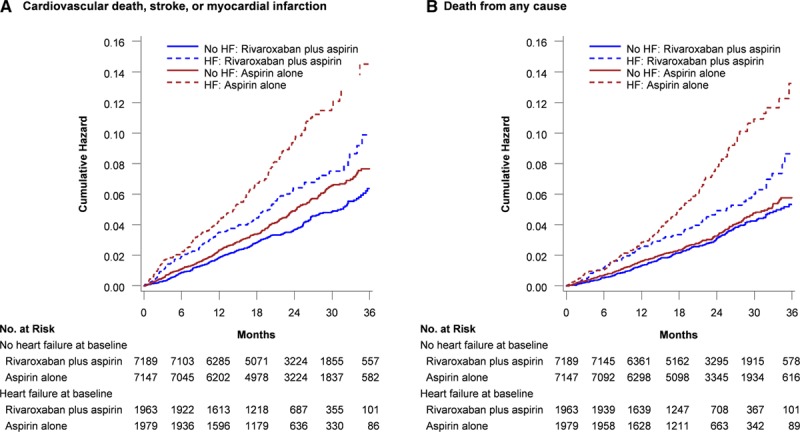
**Kaplan–Meier cumulative hazard rates.** **A**, Composite outcome of cardiovascular death, stroke, or myocardial infarction. **B**, Death from any cause. **C**, Major bleeding, by heart failure status at baseline and treatment with rivaroxaban plus aspirin or aspirin alone. Events were tabulated as time to first event. HF indicates heart failure.

Patients with a history of HF had higher rates of the primary composite of cardiovascular mortality, MI, and stroke and of total mortality than those without HF (Figure 1). Rivaroxaban plus aspirin compared with aspirin alone reduced the relative risk of the primary composite MACE outcome by 32% in patients with HF compared with 21% in those without HF (Figure 1; *P*=0.28 for interaction). The absolute risk reduction (ARR) for patients with HF was 2.4% (number needed to treat [NNT]=42) versus 1.0% (NNT=103) for those without HF. Admissions for HF in patients with baseline HF were higher than for those without HF, although the rates were similar between those treated with rivaroxaban with aspirin and aspirin alone (Table 2). Rivaroxaban plus aspirin reduced the relative risk of death of any cause by 34% in those with HF (ARR, 2.1%; NNT=48) but had a smaller effect in those without HF (Table 2; *P*=0.05 for interaction). In patients with HF, stroke occurred in 82 patients (1.4%) compared with 260 (1.2%) of those without HF. Rivaroxaban with aspirin reduced the relative risk of stroke by 52% (HR, 0.48; 95% CI, 0.28–0.83) in patients with HF and reduced stroke by 38% in those without HF (HR, 0.62; 95% CI, 0.45–0.84; *P*=0.43 for interaction). In patients with HF, MI occurred in 141 patients (2.4%) compared with 424 (2.0%) of those without HF. Rivaroxaban with aspirin numerically reduced the relative risk of MI by 23% (HR, 0.77; 95% CI, 0.51–1.15) in patients with HF compared with 11% (HR, 0.89; 95% CI, 0.71–1.13; *P*=0.5 for interaction). Rivaroxaban 5 mg BID alone compared with aspirin did not reduce the occurrence of the primary composite MACE outcomes irrespective of whether patients had a history of HF (Table 2).

**Table 2.**
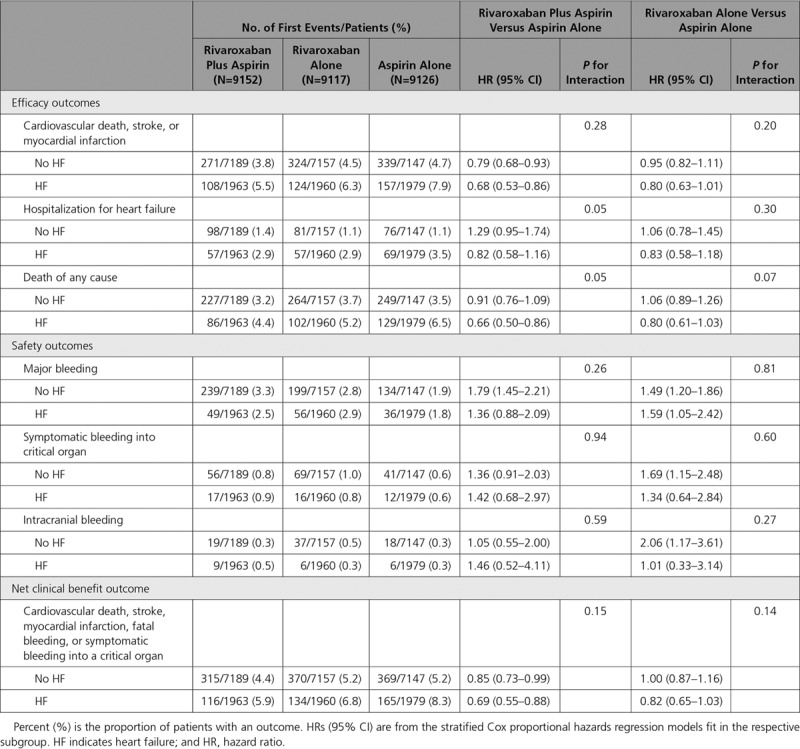
Effect of Antithrombotic Therapies According to HF Status at Baseline

Major bleeding and individual bleeding components were similar between patients with and without HF (Table 2). Major bleeding was numerically lower but not statistically different for rivaroxaban plus aspirin in patients with or without HF (Table 2; *P*=0.26 for interaction). The net clinical benefit for rivaroxaban with aspirin was positive in patients with HF (ARR 2.4%, NNT 42) and in those without HF (ARR, 0.8%; NNT=125), but these were not statistically heterogeneous (Table 2). Major bleeding was increased with rivaroxaban alone (Table 2).

### Left Ventricular EF and Outcomes

Patients with HF who had an available left ventricular EF (84% of all HF patients) predominantly had EFs ≥40% (n=4250; 72%), with fewer having EFs <40% (721; 12%; Figure I in the online-only Data Supplement). The primary MACE event rates in patients with EF <40% were 53% higher than in those with EF >40% (Table 3). The primary MACE and safety outcomes were similar according to EF category (<40%, ≥40%, EF unknown; Table 3). Other outcomes were not statistically different by treatment group and baseline EF (Table 3). Cardiac arrest occurred in 0.9% of all patients with HF and was slightly higher in those with EF <40% than in those with EF ≥40% or EF unknown [1.4% versus 0.9% versus 0.4%, respectively). Cardiac arrest rates were similar for patients with EF <40% treated with rivaroxaban with aspirin (*P*=0.94 for interaction). Incident atrial fibrillation occurred in 1.6% of patients with HF during the trial, with a higher incidence among those with EF <40% than among those with EF ≥40% and those with unknown EF (2.4% versus 1.6% versus 0.9%, respectively). There was no evidence of a treatment interaction with rivaroxaban plus aspirin by EF for major bleeding (*P*=0.47 for interaction; Table 3). There were no significant differences with rivaroxaban 5 mg BID treatment alone compared with aspirin for the primary MACE outcome if patients had a history of HF (Table 2) or by EF category (Table 3).

**Table 3.**
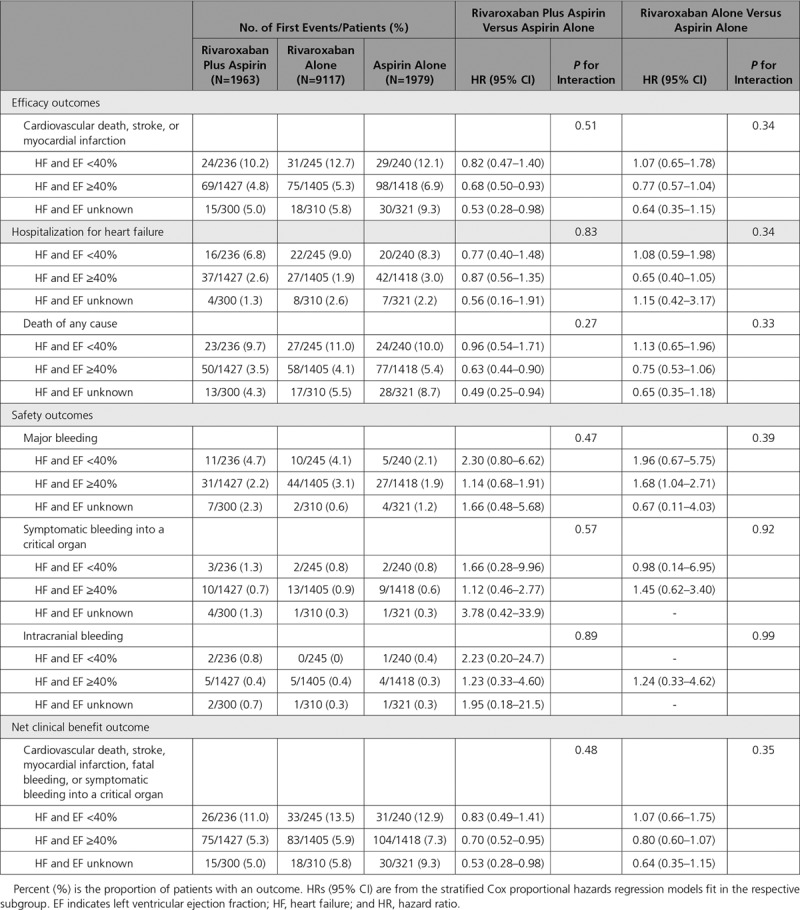
Effect of Antithrombotic Therapies in Patients With a History of HF at Baseline According to EF Categories

### Comparison of the COMPASS and COMMANDER HF Results

In patients in COMPASS with HF and EF <40%, the composite of all-cause death, MI, and stroke (the primary end point in the COMMANDER HF trial^2^) was 14.2% for aspirin and 12.7% for rivaroxaban plus aspirin, with a relative risk reduction of 13% (Table 4).

**Table 4.**
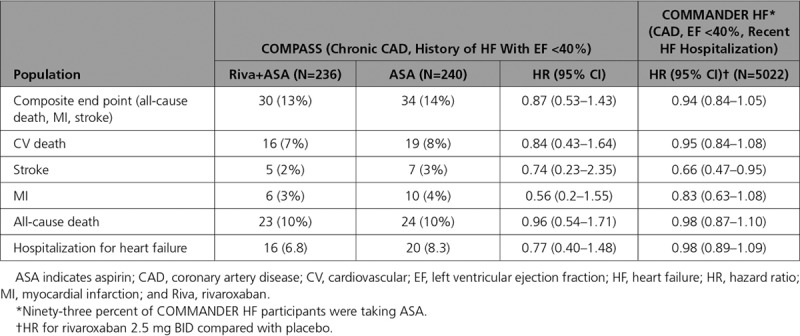
Comparison of COMMANDER HF and COMPASS Patients With HF at Baseline and EF<40%

## Discussion

In patients with chronic CAD and PAD with a history of HF at baseline, the combination of rivaroxaban and aspirin compared with aspirin alone produced similar relative risk reductions but larger ARRs in MACE and all-cause mortality compared with those who did not have HF at baseline. There was no excess in major or fatal bleeding in patients with HF. This translated into a numerically greater net clinical benefit for combination rivaroxaban and aspirin in patients with compared with those without HF. Rivaroxaban alone did not reduce the composite MACE outcome for those patients with or without HF, but it did increase major bleeding. Most patients with HF enrolled in COMPASS had preserved EF ≥40% (88%), and there were no significant differences in the effects of rivaroxaban and aspirin compared with aspirin alone in the subgroups defined by baseline EF.

In patients with chronic atherosclerotic disease, a diagnosis of HF increases the risk of MACE, hospital readmission, and mortality compared with those without HF, regardless of whether HF is related to preserved or reduced EF.^5^ Recognition of the increased risk of cardiovascular events in patients with HF informed the design of clinical trials of antithrombotic therapies in HF. Most of these trials focused on patients with HFrEF or early after MI^6–9^ and did not show a benefit of antithrombotic therapy with routine use.^6–9^ Vitamin K antagonists given alone or in combination with aspirin reduced stroke compared with aspirin alone, but the increase in bleeding negated the stroke benefit. Thus, current guidelines recommend routine antithrombotic therapy only in patients with HF at higher thromboembolic risk, such as those with left ventricular thrombus or atrial fibrillation.^10,11^ In COMPASS, we did not collect information on atrial fibrillation at randomization, although patients requiring full anticoagulation were excluded, and only 1.4% developed atrial fibrillation during the mean 23 months of follow-up. Most COMPASS patients with a history of HF had EF ≥40%, a patient population that has high risk but relatively few treatment options to improve cardiovascular outcomes.^12^ In this context, the results with the combination of rivaroxaban and aspirin may represent a worthwhile treatment option.

**Figure 2.**
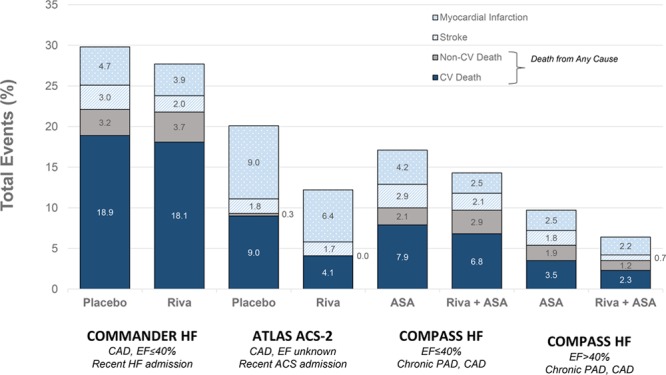
**Clinical trial events in patients with HF and CAD or PAD treated with rivaroxaban with or without aspirin.** Comparison of total event rates for primary end point, their components, and noncardiovascular death in COMMANDER HF (A Study to Assess the Effectiveness and Safety of Rivaroxaban in Reducing the Risk of Death, Myocardial Infarction, or Stroke in Participants with Heart Failure and Coronary Artery Disease Following an Episode of Decompensated Heart Failure) and in patients in the COMPASS trial (Cardiovascular Outcomes for People Using Anticoagulation Strategies) with HF, by left ventricular ejection fraction category. Multiple events could occur in a single patient. ACS indicates acute coronary syndrome; ASA, aspirin; ATLAS ACS-2, Anti-Xa Therapy to Lower Cardiovascular Events in Addition to Standard Therapy in Subjects With Acute Coronary Syndrome; CAD, coronary artery disease; CV, cardiovascular; EF, ejection fraction; HF, heart failure; PAD, peripheral artery disease; and Riva, rivaroxaban.

The COMPASS results described in the present report add incremental information to those of 2 other large trials that also tested the combination of low-dose rivaroxaban and aspirin in patients with HF, but in very different patient populations. The COMMANDER HF trial randomized patients with HFrEF (EF <40%) with recent hospitalization for acute HF decompensation to rivaroxaban versus placebo alone, with 93% on aspirin. The rate of combined all-cause mortality, MI, and stroke was 26.2% over the median 21-month follow-up, which is substantially higher than the 12.9% rate of this same outcome in COMPASS patients with EF ≤40% over a median 23-month follow-up. The most important reason for this difference was the much higher cardiovascular mortality rate in COMMANDER HF compared with COMPASS HF patients, likely driven by the acute or recently decompensated HF in COMMANDER HF compared with the chronic, stable HF cohort in COMPASS (Figure 2; Table 4). Death of patients with severe HF is commonly attributable to arrhythmia or pump failure, which may not be substantially impacted by rivaroxaban^2^ (Figure 2). Thus, rivaroxaban administration in COMMANDER HF did not significantly reduce the relative risk of the combined end point of death, MI, or stroke, which appears directionally similar to the COMPASS results in patients with EF <40% (Figure 2; Table 4). However, stroke and MI were reduced by a relative 34% and 17%, respectively, in COMMANDER HF, which is similar to the results in the COMPASS HF cohort with EF <40% (Figure 2; Table 4).^13^

Combination rivaroxaban with aspirin was also tested in the ATLAS ACS 2 trial (Anti-Xa Therapy to Lower Cardiovascular Events in Addition to Standard Therapy in Subjects With Acute Coronary Syndrome), in which patients were enrolled early after acute coronary syndrome, treated with either dual-antiplatelet therapy (93%) or aspirin (7%), and randomized to rivaroxaban 2.5 mg BID and 5 mg BID.^14^ ATLAS ACS 2 demonstrated significant reductions in MACE and all-cause mortality with low-dose rivaroxaban 2.5 mg BID, as well as an expected increase in major bleeding. In the subset of patients with HF at randomization (1694 [10.9%]), these MACE benefits were amplified, with a 41% relative risk reduction compared with placebo (16.8% to 10.1% for patients with and without HF; HR, 0.59; 95% CI, 0.42–0.81; *P*=0.002 for interaction). In addition, patients with HF had a 57% relative mortality reduction, from 9.3% to 4.1% the rivaroxaban 2.5 mg BID compared with placebo.^15^

Although the patient populations in COMMANDER HF, ATLAS ACS-2, and COMPASS were somewhat different, when taken together, the results suggest that rivaroxaban 2.5 mg BID provides antithrombotic benefits in patients with chronic HF. However, rivaroxaban appears to preferentially benefit those patients with mild to moderate HF who do not have recent decompensated HF or advanced HFrEF.

### Study Limitations

These data are based on a subgroup of patients with HF, and information on EF was incomplete. Thus, any conclusions should be viewed with appropriate caution.

### Conclusions

In patients with chronic CAD or PAD, rivaroxaban 2.5 mg BID plus aspirin as compared to aspirin alone produces similar relative risk reductions but larger absolute risk benefits in patients with mild to moderate HF who do not have recent decompensated HF or advanced HFrEF.

## Sources of Funding

The COMPASS trial was sponsored by Bayer. The authors had full control and oversight of the data, data analyses, and submission of the manuscript.

## Disclosures

Dr Branch has served on advisory boards for Janssen, Bayer, and Astra Zeneca and as a consultant for Janssen and Bayer; he has received research support from the National Institutes of Health, Population Health Research Institute, Astellas, Bayer, Sanofi, and Kestra. Dr Probstfield has received research support from Bayer. Dr Eikelboom has received grants and personal fees from Bayer, Boehringer Ingelheim, Bristol-Myers Squibb/Pfizer, Daiichi Sankyo, Janssen, Astra Zeneca, Eli Lilly, Glaxo Smith Kline, and Sanofi Aventis. Dr Bosch has received research support from Bayer. Dr Maggioni has received honoraria for participation in committees of studies sponsored by Bayer, Novartis, and Fresenius. Dr Cheng has served on advisory boards for Alnylam, Ionis. Dr Bhatt has served on advisory boards for Cardax, Elsevier Practice Update Cardiology, Medscape Cardiology, and Regado Biosciences; on the board of directors for Boston VA Research Institute, Society of Cardiovascular Patient Care, and TobeSoft; as chair of the American Heart Association Quality Oversight Committee; and on data monitoring committees for the Baim Institute for Clinical Research (formerly Harvard Clinical Research Institute, for the PORTICO trial, funded by St. Jude Medical, now Abbott), Cleveland Clinic, Duke Clinical Research Institute, Mayo Clinic, Mount Sinai School of Medicine (for the ENVISAGE trial, funded by Daiichi Sankyo), and Population Health Research Institute. Dr Bhatt has also received honoraria from the American College of Cardiology (senior associate editor, Clinical Trials and News, ACC.org; vice chair, ACC Accreditation Committee), Baim Institute for Clinical Research (formerly Harvard Clinical Research Institute; RE-DUAL PCI clinical trial steering committee funded by Boehringer Ingelheim), Belvoir Publications (editor in chief, *Harvard Heart Letter*), Duke Clinical Research Institute (clinical trial steering committees), HMP Global (editor in chief, *Journal of Invasive Cardiology*), *Journal of the American College of Cardiology* (guest editor; associate editor), Population Health Research Institute (for the COMPASS operations committee, publications committee, steering committee, and USA national coleader, funded by Bayer), Slack Publications (chief medical editor, *Cardiology Today’s Intervention*), and Society of Cardiovascular Patient Care (secretary/treasurer), WebMD (CME steering committees). Dr Bhatt has also served as deputy editor for *Clinical Cardiology*, chair of the NCDR-ACTION Registry Steering Committee, and chair of the VA CART Research and Publications Committee, and has received research funding from Abbott, Amarin, Amgen, AstraZeneca, Bayer, Boehringer Ingelheim, Bristol-Myers Squibb, Chiesi, Eisai, Ethicon, Forest Laboratories, Idorsia, Ironwood, Ischemix, Lilly, Medtronic, PhaseBio, Pfizer, Regeneron, Roche, Sanofi Aventis, Synaptic, and The Medicines Company and royalties from Elsevier (editor, *Cardiovascular Intervention: A Companion to Braunwald’s Heart Disease*). Dr Bhatt has served as site coinvestigator for Biotronik, Boston Scientific, St. Jude Medical (now Abbott), and Svelte and as trustee for the American College of Cardiology; he has also conducted unfunded research for FlowCo, Merck, Novo Nordisk, PLx Pharma, and Takeda. Dr Avezum has received personal fees from Boehringer Ingelheim. Dr Fox has received grants and personal fees from Bayer/Janssen and AstraZeneca and personal fees from Sanofi/Regeneron.

Dr Connolly has received grants from Bayer AG and personal fees from BMS, Pfizer, Portola, Boehringer Ingelheim, Servier, Daiichi Sankyo, and Medtronic.

Dr Yusuf has received grants or speaking and consulting fees, honoraria and travel expenses from Bayer, Boehringer Ingelheim, AstraZeneca, Cadila, and Ferrer. Olga Shestakovska has nothing to disclose.

## Supplementary Material
