# Supplementary material for: Rivaroxaban With or Without Aspirin in Patients With Heart Failure and Chronic Coronary or Peripheral Artery Disease: The COMPASS Trial
Source: Circulation. 2019 Jun 5;140(7):529–37. doi: 10.1161/CIRCULATIONAHA.119.039609 (PMC6693980; doi:10.1161/CIRCULATIONAHA.119.039609)

Supplemental Figure: Histogram of Available Left Ventricular Ejection Fractions for COMPASS Participants with a Baseline History of HF

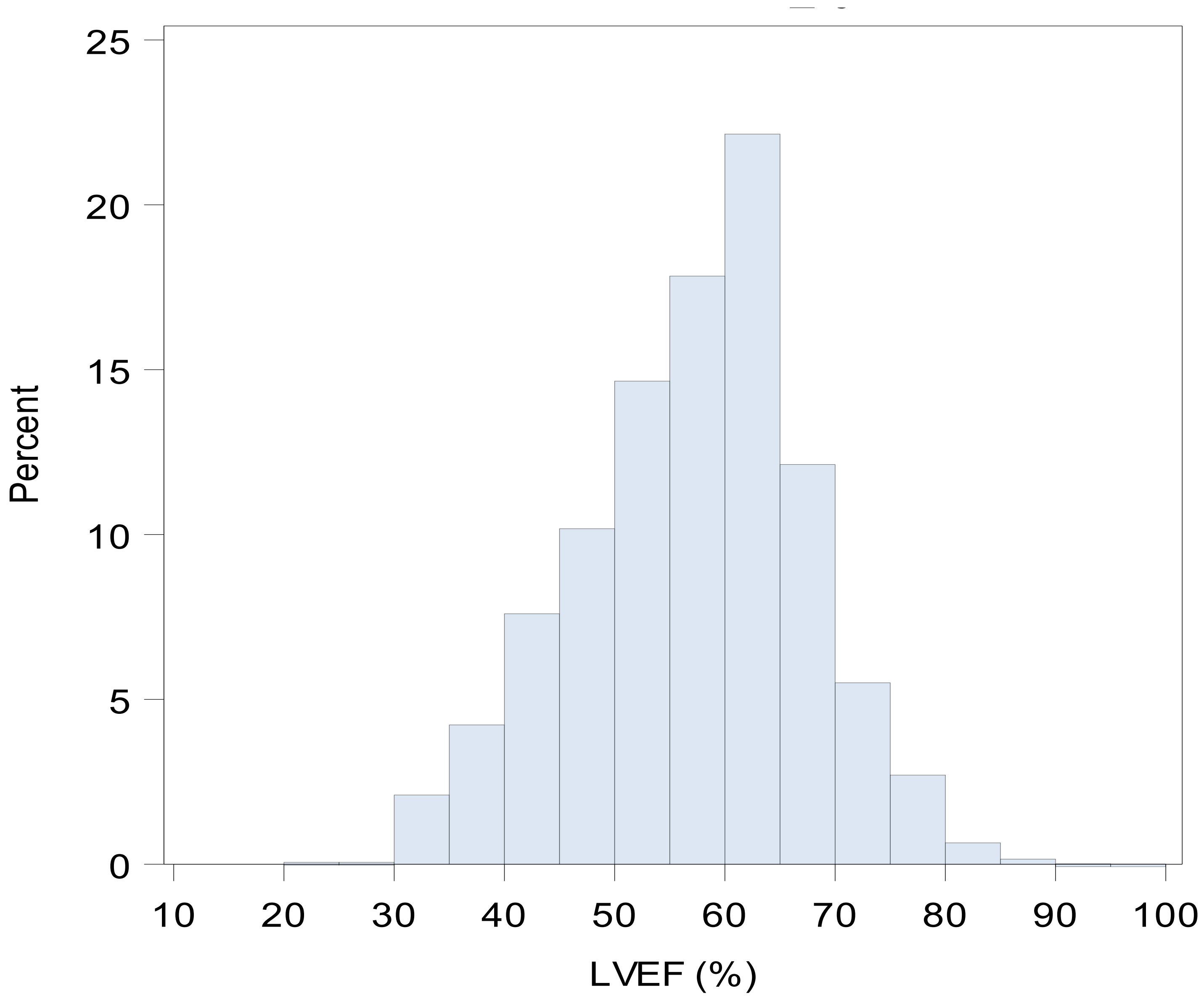

Supplement: Supplementary file 1 [file cir-140-529-s001.pdf]
